# Job vacancy at the European Centre for Disease Prevention and Control (ECDC)

**DOI:** 10.2807/1560-7917.ES.2020.25.23.2006111

**Published:** 2020-06-11

**Authors:** Eurosurveillance editorial team

**Affiliations:** 1 European Centre for Disease Prevention and Control (ECDC), Stockholm, Sweden

The European Centre for Disease Prevention and Control (ECDC) invites applications for the following position:

 

**Programme Management Officer (PHF)**

The application deadline expires on 7 Jul 2020. 

For more information, please visit the ECDC website: 

https://www.ecdc.europa.eu/en/vacancies?f[0]=deadline_date:1

